# Synergistic application of *Ascophyllum nodosum*,* Salix aegyptiaca*, and gum arabic improves salt tolerance in *Rubia tinctorum*

**DOI:** 10.1038/s41598-026-54845-x

**Published:** 2026-05-27

**Authors:** Behnam Gheisary, Ali Akbar Mozafari, Mohammad Fattahi, Davood Zaheriani

**Affiliations:** 1 Department of Horticultural Science, University of Kurdistan, Sanandaj, Iran; 2 Department of Horticultural Science, University of Urmia, Urmia, Iran; 3 Department of Plant Protection, University of Kurdistan, Sanandaj, Iran

**Keywords:** Antibacterial activity, Alizarin, Docking molecular, Synergistic effect, Biochemistry, Biotechnology, Drug discovery, Microbiology, Plant sciences

## Abstract

Salinity in arid and semi-arid regions poses a significant limitation to crop growth. The present study aims to assess the synergistic effects of *Ascophyllum nodosum*, the bark of *Salix aegyptiaca*, and gum arabic in mitigating the adverse impacts of salinity stress on *Rubia tinctorum*. The antibacterial activity of root extracts against *Ralstonia solanacearum* and *Pectobacterium carotovorum* was evaluated separately using in silico molecular docking. A simplex lattice design with 14 experimental runs was used to optimize elicitors for physiological, biochemical, and antioxidant responses under salt stress. For all measured parameters, the models exhibited high regression coefficients and were statistically significant. The optimal conditions were established at 0.19 GA, 0.27 *S. aegyptiaca,* and 0.53 *A. nodosum* for optimum in FHY 37.63 (g), DHY 4.36 (g), EL 36.13 (%), SPAD 29.97 (%), RWC (%) 88.38, proline 1.91 (mg/g FW), MDA 5.21 (µmol/g FW), TPC 70.41 (mg GA/g FW), TFC 20.43 (mg Qu/g FW), PAL 3.90 (µmol cinnamic acid/g FW), DPPH scavenging activity 82.08 (%), GPX 0.78 (U min^-1^ g^-1^ FW), CAT 5.60 (U min^-1^ g^-1^ FW), APX 5.91 (U min^-1^ g^-1^ FW), alizarin 1.41(mg/g). The results indicated that the root extract effectively inhibited the growth of both bacteria compared to the control. In *R. solanacearum* and *P. carotovorum,* PDB: 5NMP and 4ZA2 exhibited notable affinities with ligand combinations, respectively. The synergistic models enhanced the potential effectiveness of the treatments and can help *R. tinctorum* reduce the adverse effects of salinity stress. The optimized formulation remains promising, offering significant and strategic potential for mitigating the adverse effects of salinity stress.

## Introduction

One of the major challenges in agricultural production across many regions of the world is soil salinity^1^. In higher plants, soil salinity leads to ionic toxicity and osmotic stress, which cause disturbances in the uptake of nutrients and water by roots, resulting in reduced photosynthesis, growth, and overall plant productivity^2^. Among the metabolic adjustments developed by plants to cope with salt stress are cellular regulation mechanisms and increased synthesis of sugars, proline, and glutamate, which help in osmotic adjustment and protection of cellular structures^3^. Another consequence of salinity stress is the excessive production and accumulation of reactive oxygen species (ROS), which lead to oxidative damage in cellular membranes and biomolecules^4^. The negative effects of salinity on growth (*Oenanthe javanica*)^5^, chlorophyll content (*Phaseolus vulgaris*)^6^, nitrogen assimilation (*Fagopyrum tataricum* Gaertn)^7^ , and enzyme activity (*Moringa oleifera* Lam)^8^ have been reported. Mitigating the detrimental effects of salinity on plants remains a key focus of plant science research. The application of natural elicitors, including *Ascophyllum nodosum***,** willow (Salix) (*Salix aegyptiaca*) extracts, and gum arabic, can emerge as a sustainable and cost-effective strategy to reduce the impacts of salt stress and improve plant performance under adverse conditions.

The extract of *A. nodosum* serves as a natural biofertilizer, enhancing plant resistance to abiotic stresses, particularly salinity and drought^9,10^. *A. nodosum* extract can alleviate salt stress by improving nutrient transport and ionic balance within plant tissues^11^. Furthermore, *A. nodosum* enhances the biosynthesis of secondary and biochemical metabolites, thereby strengthening the plant’s physiological and metabolic resistance to stress conditions^12^.

Musk willow (*Salix aegyptiaca*), also known as the Persian willow, belongs to the Salicaceae family and is native to the Middle East. The bark and leaves of *S. aegyptiaca* have been used in traditional medicine for their beneficial effects^13^. The extract of *S. aegyptiaca* is rich in salicylic acid and other polyphenolic compounds, including caffeic acid, gallic acid, vanillin, catechin, and quercetin^14^. Salicylic acid can activate endogenous acquired resistance signals in plants under stress conditions. Li et al. (2014)^15^ reported that salicylic acid increased chlorophyll content, proline levels, and antioxidant enzyme activity in *Torreya grandis* under salt stress.

Gum Arabic (GA), a natural polysaccharide derived from the Acacia tree, is widely used in a range of commercial and industrial applications. This GA offers high performance and low cost, making it valuable in the pharmaceutical, food, adhesive, and cosmetic industries. GA composition generally includes nitrogen, ash, uronic acid, arabinogalactan, and glycoproteins^16,17^. Arabinose increased salt stress tolerance in *Arabidopsis* by maintaining cell wall integrity under saline conditions^18^.

Madder (*Rubia tinctorum* L.) is a perennial herb in the madder family (Rubiaceae). The leaves and roots of *R. tinctorum* are used in food, cosmetics, and the pharmaceutical industry^19^. Leaves and roots of *R. tinctorum* are utilized for medicinal purposes, including antifungal, antioxidant, anti-inflammatory, antimicrobial, and treating skin diseases treatments^20,21^. The red color of the rhizomes and roots of *R. tinctorum* is caused by alizarin, which is used as a natural dye^22^. Alizarin, as a colorimetric indicator, can be applied in the food industry and food packaging, and is regarded as less hazardous than synthetic chemical substances^23^.

Medicinal plants are valuable resources for managing plant diseases, particularly those caused by bacterial pathogens^24^. Alizarin possesses antibacterial and antioxidant activity, which can inhibit the growth of phytopathogenic bacteria, such as *Ralstonia solanacearum and Pectobacterium carotovorum*. *R. solanacearum* is a soil-borne pathogen that causes vascular wilt disease in various plant hosts, particularly in tomato^25^. *P. carotovorum* is a Gram-negative pathogen responsible for soft rot disease through the degradation of plant cell walls in a wide range of hosts^26^.

Drug design for the biochemical and biological control of bacteria is of great importance, and molecular docking is an effective tool in this regard. Molecular docking is a cost-effective, efficient, and precise computational approach used to determine the structural mechanisms of target–ligand interactions at the molecular level^27^. In this method, the ligand (alizarin) 's binding affinity and potential interaction energy with bacterial target proteins are evaluated. The novelty and innovation of this study primarily lie in applying synergistic effects using a lattice design to investigate the impact of *A. nodosum*, *S. aegyptiaca*, and GA on the physiological, biochemical, and antioxidant activity of *R. tinctorum* under salinity stress. To the best of our knowledge, no prior research has investigated the synergistic effect of three elicitors on plants under stress. Furthermore, this research highlights the significance of alizarin in controlling key plant pathogenic bacteria via multi-ligand molecular docking.

## Material and methods

### Plant material, growth conditions, and salinity treatments

Seeds of *Rubia tinctorum* were obtained from Pakan Bazr Company (Isfahan, Iran). The seeds were surface-sterilized with 70% ethanol (30 s) and then with 1% sodium hypochlorite for 5 min. The seeds were planted in plastic pots filled with a clay loam mixture (2:3, v/v). The soil used in this experiment had a pH of 7.82, an EC of 0.79 dS/m, an available potassium of 189 mg kg^−1^, an available phosphorus of 7.02 mg kg^−1^, a total nitrogen of 0.14%, and a CaCO_3_ of 32.1%. The pots were maintained in a greenhouse under natural light, with a temperature range of 21 ± 2 °C to 28 ± 2 °C and a relative humidity of 55–65%. After germination and seedling emergence, ten plants per pot were selected for retention.

### Factors and experimental matrix for mixture design

Initially, *R. tinctorum* plants were exposed to different salinity levels (0, 25, 50, and 100 mM NaCl). Based on physiological and biochemical measurements (unpublished data), 50 mM NaCl was selected as the optimal salinity level for the main experiment. Subsequently, treatments with GA, *S. aegyptiaca*, and *A. nodosum* under 50 mM NaCl salinity stress were designed using a simplex lattice design. The *A. nodosum*, containing 2.5% total nitrogen (N), 3% potassium (K₂O), 10.5% fulvic acid, and 15% organic matter, was obtained from Hamyar Dasht Abaron Company (Tehran, Iran). To prepare the *S. aegyptiaca* bark extract, 100 g of bark collected from the campus of Urmia University, Iran (45° 03′ 25″ E, 37° 38′ 31″ N), was ground into fine powder. The powdered samples were soaked in 1 L of distilled water for 24 h, then filtered. The resulting solution was extracted using a Soxhlet apparatus for 5 h. After cooling, the obtained extract was filtered and subsequently diluted to the desired concentrations for further use.

Mr. Hossein Maroufi, an agricultural engineer at the Agricultural and Natural Resources Research Center of Kurdistan, Sanandaj, Iran, taxonomically identified the plant species. A voucher specimen, *S. aegyptiaca* (No. 1510), was prepared and deposited in the herbarium of the Department of Horticulture at Urmia University. Sampling was carried out in accordance with all relevant institutional, national, and international guidelines for plant collection from the outskirts of Sanandaj city, following the IUCN Policy Statement on Research Involving Species at Risk of Extinction and the Convention on International Trade in Endangered Species of Wild Fauna and Flora (CITES).

Furthermore, the GA powder (Sigma-Aldrich) was dissolved in distilled water on a heating block at 40 °C with continuous stirring until a clear, transparent solution was obtained. After cooling, glycerol (Sigma-Aldrich) was added to the solution as a plasticizer at 1.5%.

A total of 6 points and 14 experiments were conducted, with three replications for each of the three components: gum arabic (X_1_), *S. aegyptiaca* (X_2_), and *A. nodosum* (X₃). The treatments ranged from 0 to 1 in the mixture, with their total equal to 1. The mixtures (4% v/v) were applied at the 8-leaf stage of the plant under salt stress (50 mM NaCl) by foliar spraying at six-day intervals, three times. The salt concentration in the irrigation water was gradually increased, and the final concentration was reached after three successive applications. A quadratic model for the independent variables and response was formulated as follows:

$$Y = \, \Sigma_{{1}} {\mathrm{X}}_{{1}} + \, \Sigma_{{2}} {\mathrm{X}}_{{2}} + \, \Sigma_{{3}} {\mathrm{X}}_{{3}} + \, \Sigma_{{{12}}} {\mathrm{X}}_{{1}} {\mathrm{X}}_{{2}} + \, \Sigma_{{{13}}} {\mathrm{X}}_{{1}} {\mathrm{X}}_{{3}} + \, \Sigma_{{{23}}} {\mathrm{X}}_{{2}} {\mathrm{X}}_{{3}}$$

where *Y* represents the dependent variable, and *Xi* denotes the independent variable; *Σ*_1_, *Σ*_2_, and *Σ*_3_ correspond to the coefficients of the linear terms; *Σ*_*12*_, *Σ*_13_, and *Σ*_23_ represent the coefficients of the binary terms; and ε is the error term.

### Biomass and Spad value chlorophyll content

The aerial parts of the plants (10 plants per pot) were harvested after two months. The fresh herb yield (FHY) was recorded, and the samples were then placed in an oven at 72 °C for 48 h to determine the dry herb yield (DHY) of each plan. The Spad value chlorophyll content (SPAD) was measured using the Minolta SPAD-502 Chlorophyll Meter device.

### Relative water content (RWC)

For measuring RWC, leaf discs with a diameter of 8 mm were prepared, and their fresh weight (FW) was immediately recorded. The discs were then floated in distilled water at 4 °C for 4 h. Afterward, they were removed, gently blotted dry with filter paper, and their turgid weight (TW) was measured. Subsequently, the discs were oven-dried at 70 °C for 48 h, and their dry weight (DW) was recorded^28^. The RWC was calculated using the following formula:

$${\text{RWC }}\left( \% \right) \, = \, \left[ {\left( {{\mathrm{FW}} - {\mathrm{DW}}} \right)/\left( {{\mathrm{TW}} - {\mathrm{DW}}} \right)} \right] \, \times \, 100$$

### SPAD and electrolyte leakage (EL)

The SPAD value was recorded using a Minolta SPAD-502 chlorophyll meter^29^.

The membrane stability index in madder leaves was determined by measuring electrolyte leakage (EL)^30^. Leaf discs (8 mm in diameter) were placed in 12 mL of distilled water and shaken for 24 h. The initial electrical conductivity (EC₁) was then measured. Subsequently, the samples were autoclaved for 20 min, and after cooling, the electrical conductivity (EC₂) of the solution was measured. Finally, EL was calculated using the following formula.

$${\mathrm{EL}}\left( \% \right) \, = \, \left( {{\mathrm{EC1}}/{\mathrm{EC2}}} \right) \, \times { 1}00$$

### Malondialdehyde (MDA) content

For this purpose, fresh leaves (0.2 g) were homogenized with trichloroacetic acid (5 mL of 0.1%) (TCA) and centrifuged (Sigma, Germany) for 10 min. Subsequently, 2 mL of TCA (20%) was added to thiobarbituric acid (0.5%). The mixture was then heated at 95°C in a water bath for 30 min. After that, absorbance measurements were recorded at 532 and 600 nm wavelengths using a spectrophotometer^31^.

### Free proline content

The leaves were extracted using 5 mL of 90% ethanol and centrifuged at 3500 rpm for 10 min. Briefly, 10 mL of distilled water and 5 mL of glacial acetic acid were added to 1 mL of the ethanol extract, followed by the addition of 5 mL of the ninhydrin reagent and thorough mixing. The solution was then incubated for 45 min at 100 °C. Afterward, 10 mL of benzene was added. The absorbance was determined by a spectrophotometer at 516 nm^32^.

### Phenylalanine ammonia-lyase (PAL) activity

Leaf samples of *R. tinctorum* (0.2 g) were homogenized in 2 mL of buffer solution containing 15 mM β-mercaptoethanol and 50 mM Tris–HCl (pH 8.5). The homogenate was then centrifuged at 4000 rpm for 10 min. To the supernatant, 20 µL of 500 mM Tris–HCl (pH 8) and 6 mM L-phenylalanine were added. Subsequently, 50 µL of 5N HCl was added to the reaction mixture, and the absorbance was measured at 290 nm using a spectrophotometer. Cinnamic acid was used to prepare the standard calibration curve.

### Preparation of extracts

Dried leaf samples (0.5 g) were homogenized in 20 mL of 80% methanol using a mortar and pestle. The obtained extracts were centrifuged at 10,000 rpm for 5 min and then placed in an ultrasonic bath at 35 °C for 20 min. The resulting solutions were filtered and stored at 4 °C until analysis for total phenolic content, total flavonoid content, and DPPH scavenging activity.

### Total phenolic content (TPC)

For this purpose, 40 µL of the extract was mixed with 1 mL of 10% Folin–Ciocalteu reagent and 180 µL of distilled water. The mixture was allowed to stand at room temperature for 10 min. Then, 480 µL of 10% (w/v) sodium carbonate solution was added, and the mixture was incubated in the dark at 25 °C for 30 min. The absorbance was measured at 760 nm using a spectrophotometer^33^.

### Total flavonoid content (TFC)

For the determination of TFC, 30 μL of extract was mixed with 150 μL of sodium nitrite (5%) and 300 μL of aluminum chloride (10%). Then, 1 cc NaOH (1 M) was added after 5 min and incubated in a dark place (30 min). Finally, the absorbance was measured at 510 nm using a spectrophotometer^34^.

### DPPH scavenging activity

For the determination of antioxidant activity, 30 µL of the extract was added to 2 mL of DPPH solution (20 µg·mL⁻^1^) and incubated in the dark at room temperature for 25 min. The absorbance of the mixture was then measured at 515 nm using a spectrophotometer^35^. The antioxidant activity was calculated using the following equation:

$${\text{Antioxidant activity}} = \, \left[ {\left( {{\text{A control}} - {\text{A sample}}} \right) \, \div {\text{A control}}} \right] \, \times { 1}00$$

### Measurement of antioxidant enzyme activity

For the determination of antioxidant enzyme activity, 0.3 g of fresh leaves and roots was homogenized with 2 mL potassium phosphate buffer (pH 6.8, 50 mM) containing 0.05% PVP (polyvinylpyrrolidone), HCl-Tris (ethylenediaminetetraacetic acid) (0.5%), for 10 min at 4°C and subjected to centrifuging (13,000 rpm, 20 min). The activities of ascorbate peroxidase (APX), catalase (CAT), and glutathione peroxidase (GPX) were measured according to the same procedures reported by^36^, Aebi (1984)^37^, and Iskusnykh et al. (2013)^38^, respectively.

### Alizarin

For this purpose, 0.1 g of root was extracted using 80% ethanol and 3 mL of 3 N HCl, followed by incubation in a 70 °C water bath for 2 days. The resulting extracts were centrifuged at 4000 rpm for 30 min. Subsequently, the samples were measured at a wavelength of 517 nm using a spectrophotometer^39^.

### Antibacterial

The antibacterial activity of the alizarin extract against bacteria *Ralstonia solanacearum* and *Pectobacterium carotovorum* was evaluated using the Kirby–Bauer disk diffusion method. For this purpose, a bacterial suspension with an optical density of 0.13–0.08 at 600 nm was prepared and spread evenly on nutrient agar plates. Sterile paper disks impregnated with the extract at concentrations of 400, 600, and 1000 µg/mL were placed on the agar surface. Ampicillin (50 µg/mL) and distilled water were used as positive and negative controls, respectively. The plates were incubated at 30 °C for 24 h, after which the inhibition zone diameters were measured using a digital caliper^40^.

### Docking molecule

The interaction between the receptor and the ligand was analyzed using AutoDock Vina version 1.1.2. The receptor structure was obtained from the Protein Data Bank (PDB) (https://www.rcsb.org/), and the ligand structure was downloaded from PubChem (https://pubchem.ncbi.nlm.nih.gov/compound/479503). AutoDock Tools version 1.5.6 was employed to prepare the ligand and receptor structures for docking. Molecular interactions between ligands and protein binding sites were further analyzed using Discovery Studio Client version 4.5. In this study, bacterial proteins from *R. solanacearum* (5NMP, 4FDB, and 1UXQ) and *P. carotovorum* (1N4X, 4UBU, and 4ZA2), along with the ligand alizarin, were selected for molecular docking analysis.

### Statistical analysis

The experimental design (14 runs) and statistical analyses were conducted using Design-Expert software (Version 13.0.0, Stat-Ease, Inc., Minneapolis, MN, USA). A quadratic model was used for validation; *R*^*2*^ and adjusted *R*^*2*^ (*R*^*2*^ adj) values were used to evaluate the model’s quality. The *F*-ratio was used to determine the statistical significance of the model terms. The accuracy of the mathematical model was assessed using the lack-of-fit test and a significance level of *P* < 0.05. Correlation and PCA analyses were performed using RStudio 4.1.3 (TRL: http://www.rstudio.com/).

## Result and discussion

### Statistical validation of the model

The combinations of treatments, along with the measured responses and the predicted values, are presented in Table 1. According to Table 2, the analysis of variance (quadratic model) indicates that the main regression model was statistically significant for all responses. These results demonstrate a strong correlation between the predicted values and the mathematical models. The predictive performance of the models was evaluated using the coefficient of determination *R*^*2*^ and the adjusted *R*^*2*^. The following equations describe the variability in the obtained data.

**Table 1:** Matrix design, results, and predicted value of the experiment for the effect of gum arabic, *S. aegyptiaca*, and *A. nodosum* on some phytochemical parameters of madder (*R. tinctorum*) under 50 mM NaCl concentrations.

Run	X_1_	X_2_	X_3_	FHY (g plant^-1^)		DHY (g plant^-1^)		RWC (%)		EL (%)		SPAD (%)		MDA (µmol/g FW)		Proline (mg/g FW)		PAL (µmol Cin/g FW)		TPC (mg GA/g FW)		TFC (mg Qu/g FW)		DPPH (%)		GPX (U min^-1^ g^-1^ FW)		CAT (U min^-1^ g^-1^ FW)		APX (U min^-1^ g^-1^ FW)		Alizarin (mg/g)	
				Obs	Pre	Obs	Pre	Obs	Pre	Obs	Pre	Obs	Pre	Obs	Pre	Obs	Pre	Obs	Pre	Obs	Pre	Obs	Pre	Obs	Pre	Obs	Pre	Obs	Pre	Obs	Pre	Obs	Pre
1	0	1	0	35.90	34.98	2.87	2.70	74.05	71.58	45	43.6	29.41	28.51	5.43	5.09	2.02	1.89	2.98	3.37	65.53	65.68	21.54	20.2	65.43	71.38	1.1	1.07	3.21	3.04	5.43	5.52	1.23	1.18
2	0.5	0.5	0	34.49	33.65	4.04	3.95	82.48	82.77	28	27.4	33.41	33.05	7.3	6.85	2.74	2.36	3.03	3.24	55.46	49.59	16.31	16.7	68.87	64.37	0.91	0.88	4.32	4.19	3.87	4.67	2.05	2.10
3	0	0	1	40.61	42.29	6.09	5.49	86.00	88.32	35	31.6	32.11	32.54	7.65	7.01	1.7	1.64	2.88	2.83	43.23	46.50	17.54	17.24	69.39	74.04	0.65	0.59	3.98	3.92	8.65	8.43	1.22	1.39
4	0.5	0	0.5	29.01	27.84	3.22	2.87	90.31	94.88	44	45.0	27.51	27.20	6.1	5.89	1.18	1.05	4.12	4.32	81.61	86.47	24.32	24.23	59.43	64.65	0.95	1.06	3.55	3.57	3.54	3.97	1.17	1.19
5	0.166	0.166	0.666	33.84	37.16	2.98	4.42	91.23	84.44	38	36.8	29.61	30.00	4.87	5.55	1.47	1.74	3.89	3.79	66.92	69.29	20.43	20.90	91.06	79.99	0.8	0.77	4.89	5.16	6.43	6.20	1.21	1.35
6	0.166	0.666	0.166	37.28	36.66	4.14	3.85	70.57	74.82	33	35.0	31.11	30.60	5.07	5.22	2.4	2.26	3.8	3.61	55.30	63.38	20.43	19.08	80.43	79.01	0.88	0.87	6.01	5.07	6.23	5.46	1.95	1.58
7	0.333	0.333	0.333	38.03	34.28	4.22	3.91	85.99	82.59	40	35.4	29.61	30.16	5.55	5.49	1.77	1.98	4.11	3.92	80.84	70.32	18.54	20.4	72.01	76.77	1.02	0.87	5.32	5.25	6.01	4.95	1.6	1.58
8	0.666	0.166	0.166	30.76	33.97	4.01	3.73	89.36	88.20	31	33.8	30.01	29.87	6.4	6.18	1.78	1.60	4.05	3.92	72.30	67.49	19.43	19.5	67.43	62.33	1.01	0.93	4.32	4.28	4.07	4.55	1.81	1.74
9	0	0.5	0.5	46.19	46.29	5.54	5.19	67.05	69.82	32	33.2	30.41	30.22	4.55	4.22	2.32	2.19	3.55	3.77	63.53	64.10	19.13	19.5	88.32	92.32	0.54	0.62	6.43	6.75	6.72	7.57	1.26	1.38
10	1	0	0	42.57	42.37	4.38	4.66	82.48	86.65	27	28.4	30.11	29.34	6.23	6.28	0.82	1.03	3.66	3.75	56.61	55.64	15.43	17.0	48.14	49.01	0.76	0.82	4.55	3.85	5.54	6.37	1.84	1.84
11	0.5	0.5	0	32.66	33.65	3.62	3.95	82.18	82.77	26.9	27.4	32.71	33.05	6.43	6.85	1.97	2.36	3.22	3.24	40.76	49.59	17.43	15.79	57.86	64.37	0.76	0.88	3.65	4.19	4.87	4.67	2.01	2.10
12	0	1	0	33.61	34.98	2.37	2.70	71.32	71.58	43	43.6	27.41	28.51	4.74	5.09	1.7	1.89	3.77	3.37	69.07	65.68	18.54	20.2	78.29	71.38	1.08	1.07	2.65	3.04	5.54	5.52	1.24	1.28
13	1	0	0	43.01	45.37	4.77	4.66	91.03	86.65	31	28.4	28.51	29.34	6.21	6.28	1.18	1.03	3.88	3.75	53.61	55.64	16.87	17.0	49.60	49.01	0.89	0.82	3.23	3.85	7.54	6.37	1.86	1.84
14	0	0	1	44.84	42.29	5.31	5.49	89.17	88.32	28	31.6	33.08	32.54	6.54	7.01	1.67	1.64	2.83	2.83	51.12	46.50	16.54	17.2	76.43	74.04	0.55	0.59	4.05	3.92	8.32	8.43	1.66	1.39

*X1* Gum Arabic, *X2 Salix aegyptiaca*, *X3* *Ascophyllum nodosum*, *Obs* observed, *Pre* prediction, *FHY* Fresh Herb Yield, *DHY* Dry Herb Yield, *SPAD* Spad value chlorophyll content, *RWC* Relative Water Content, *EL* Electrolyte Leakage, *MDA* Malondialdehyde, *TPC* Total Phenolic Content, *TFC* Total Flavonoid Content, *DPPH* 2,2-diphenyl-1-picrylhydrazyl, *PAL* Phenylalanine Ammonia-Lyase, *GPX* Guaiacol Peroxidase, *CAT* Catalase Peroxidase, *APX* Ascorbate Peroxidase.

**Table 2:** Variance analysis (ANOVA) for the quadratic models.

	FHY					DHY					RWC				
SOV	SS	df	MS	*F-*value	*P*-value	SS	df	MS	*F-*value	*P*-value	SS	df	MS	*F-*value	*P*-value
Quadratic model	329.0	5	65.81	9.73	0.0019**	11.50	5	2.30	5.55	0.0169**	748.36	5	149.6	7.66	0.0074**
Residual	46.98	8	5.87			3.32	8	0.414			154.8	8	19.35		
Lack of fit	33.66	4	8.42	1.32	0.19^ns^	2.73	4	0.6823	4.66	0.0826^ns^	109.4	1	27.36	2.41	0.207^ns^
*R*^2^	0.875					0.776					0.828				
*A. R*^2^	0.797					0.636					0.721				
*Pre. R*^*2*^	0.68					0.363					0.269				

	EL					SPAD					MDA				
SOV	SS	df	MS	*F-*value	*P*-value	SS	df	MS	*F-*value	*P*-value	SS	df	MS	*F-*value	*P*-value
Quadratic model	474.3	5	94.84	10.42	0.0024**	43.42	5	8.86	14.21	0.0008**	9.51	5	1.90	7.83	0.006**
Residual	72.70	8	9.09			4.89	8	0.611			1.94	8	0.243		
Lack of fit	37.60	4	9.40	1.07	0.473^ns^	0.893	4	0.223	0.223	0.911^ns^	0.711	4	0.177	0.577	0.6963^ns^
*R*^2^	0.866					0.898					0.830				
*A. R*^2^	0.783					0.835					0.724				
*Pre. R*^*2*^	0.595					0.636					0.379				

	Proline					PAL					TPC				
SOV	SS	df	MS	*F-*value	*P*-value	SS	df	MS	*F-*value	*P*-value	SS	df	MS	*F-*value	*P*-value
Quadratic model	2.85	5	0.570	7.41	0.0071**	2.34	5	0.468	6.63	0.01**	1643	5	328.7	6.75	0.0095**
Residual	0.615	8	0.077			0.56	8	0.070			389.7	8	48.71		
Lack of fit	0.206	4	0.051	0.503	0.738^ns^	0.209	4	0.052	0.588	0.689^ns^	239.9	4	59.79	1.60	0.329^ns^
*R*^2^	0.822					0.805					0.808				
*A. R*^2^	0.711					0.684					0.688				
*Pre. R*^*2*^	0.414					0.093					0.438				

TFC						DPPH					GPX				
SOV	SS	df	MS	*F-*value	*P*-value	SS	df	MS	*F-*value	*P*-value	SS	df	MS	*F-*value	*P*-value
Quadratic model	61.64	5	12.33	8.14	0.005**	1789	5	357.9	7.33	0.007**	0.350	5	0.07	6.93	0.0088**
Residual	12.11	8	1.51			390	8	48.84			0.080	8			
Lack of fit	5.45	4	1.36	0.57	0.575^ns^	221.5	4	55.39	1.31	0.399^ns^	0.050	4	0.01	2.25	0.226^ns^
*R*^2^	0.835					0.82					0.812				
*A. R*^2^	0.733					0.70					0.695				
*Pre. R*^*2*^	0.461					0.34					0.052				

			CAT				APX				Alizarin				
SOV	SS	df	MS	*F-*value	*P*-value	SS	df	MS	*F-*value	*P*-value	SS	df	MS	*F-*value	*P*-value
Quadratic model	12.67	5	2.53	8.29	0.005**	26.05	5	5.21	7.30	0.007**	1.24	5	0.248	7.13	0.008**
Residual	2.45	8	0.305			5.71	8	5.73	8.02		0.278	8	0.034		
Lack of fit	1.19	4	0.297	0.949	0.519^ns^	3.15	4	0.714	1.23	0.422^ns^	0.181	4	0.045	1.86	0.280^ns^
*R*^2^	0.83					0.82		0.64			0.816				
*A. R*^2^	0.737					0.70					0.702				
*Pre. R*^*2*^	0.488					0.22					0.460				

ns, and ** are non-significant, significant at *p* ≤ 0.001 respectively, *SOV* source of variation, *SS* sum of square, *MS* mean square, *Df* degree of freedom, *R*^*2*^ Coefficient of determination, *A. R*^*2*^ adjusted *R*^*2*^, *Pre R*^*2*^* predicted R*^*2*^* and.* *FHY* Fresh Herb Yield, *DHY* Dry Herb Yield, *SPAD *Spad value chlorophyll content, RWC Relative Water Content, EL Electrolyte Leakage, MDA Malondialdehyde, TPC Total Phenolic Content, *TFC* Total Flavonoid Content, *DPPH* 2,2-diphenyl-1-picrylhydrazyl, *PAL* Phenylalanine Ammonia-Lyase, *GPX* Guaiacol Peroxidase, *CAT* Catalase, *APX* Ascorbate Peroxidase.

$${Y}_{ \mathrm{F}\mathrm{H}\mathrm{Y} }=+45.38{X}_{1}+34.99{X}_{2}+42.29{X}_{3}-26.10{X}_{1}{X}_{2}-63.97{X}_{1}{X}_{3}+\mathrm{30.64.57}{X}_{2}{X}_{3}$$

$${Y}_{ \mathrm{D}\mathrm{H}\mathrm{Y} }=+4.66{X}_{1}+2.70{X}_{2}+5.50{X}_{3}+1.07{X}_{1}{X}_{2}-8.81{X}_{1}{X}_{3}+4.38{X}_{2}{X}_{3}$$

$${Y}_{ \mathrm{E}\mathrm{L}}=+ 28.48{X}_{1}+43.62{X}_{2}+31.65{X}_{3}-34.29{X}_{1}{X}_{2}+59.77{X}_{1}{X}_{3}-17.37{X}_{2}{X}_{3}$$

$${Y}_{ \mathrm{S}\mathrm{P}\mathrm{A}\mathrm{D}}=+ 29.35{X}_{1}+28.51{X}_{2}+32.55{X}_{3}+16.50{X}_{1}{X}_{2}-14.98{X}_{1}{X}_{3}-1.21{X}_{2}{X}_{3}$$

$${Y}_{ \mathrm{R}\mathrm{W}\mathrm{C}}=+ 86.66{X}_{1}+71.58{X}_{2}+88.33{X}_{3}+14.62{X}_{1}{X}_{2}+29.58{X}_{1}{X}_{3}-40.53{X}_{2}{X}_{3}$$

$${\mathrm{Y}}_{ \mathrm{P}\mathrm{r}\mathrm{o}\mathrm{l}\mathrm{i}\mathrm{n}\mathrm{e}}=+ 1.04{X}_{1}+1.90{X}_{2}+1.65{X}_{3}+3.60{X}_{1}{X}_{2}-1.15{X}_{1}{X}_{3}+1.68{X}_{2}{X}_{3}$$

$${Y}_{ \mathrm{M}\mathrm{D}\mathrm{A}}=+ 6.29{X}_{1}+5.09{X}_{2}+7.01{X}_{3}+4.65{X}_{1}{X}_{2}-3.02{X}_{1}{X}_{3}-7.32{X}_{2}{X}_{3}$$

$${Y}_{ \mathrm{T}\mathrm{P}\mathrm{C}}=+ 55.65{X}_{1}+65.69{X}_{2}+46.51{X}_{3}-44.29{X}_{1}{X}_{2}+141.61{X}_{1}{X}_{3}+32.03{X}_{2}{X}_{3}$$

$${Y}_{ \mathrm{T}\mathrm{F}\mathrm{C}}=+ 17.08{X}_{1}+20.22{X}_{2}+17.25{X}_{3}-11.40{X}_{1}{X}_{2}+28.30{X}_{1}{X}_{3}+3.27{X}_{2}{X}_{3}$$

$${Y}_{ \mathrm{P}\mathrm{A}\mathrm{L}}=+ 3.76{X}_{1}+3.37{X}_{2}+2.84{X}_{3}-1.30{X}_{1}{X}_{2}+4.09{X}_{1}{X}_{3}+2.66{X}_{2}{X}_{3}$$

$${Y}_{ \mathrm{D}\mathrm{P}\mathrm{P}\mathrm{H}}=+ 49.01{X}_{1}+71.39{X}_{2}+74.05{X}_{3}+16.69{X}_{1}{X}_{2}+12.51{X}_{1}{X}_{3}+78.41{X}_{2}{X}_{3}$$

$${Y}_{ \mathrm{G}\mathrm{P}\mathrm{X}}=+ 0.82{X}_{1}+1.08{X}_{2}+0.59{X}_{3}-0.24{X}_{1}{X}_{2}+1.43{X}_{1}{X}_{3}-0.81{X}_{2}{X}_{3}$$

$${Y}_{ \mathrm{C}\mathrm{A}\mathrm{T}}=+ 3.85{X}_{1}+3.04{X}_{2}+3.93{X}_{3}+3.00{X}_{1}{X}_{2}-1.26{X}_{1}{X}_{3}+13.08{X}_{2}{X}_{3}$$

$${Y}_{ \mathrm{A}\mathrm{P}\mathrm{X}}=+ 6.37{X}_{1}+5.53{X}_{2}+8.44{X}_{3}-5.08{X}_{1}{X}_{2}-13.70{X}_{1}{X}_{3}+2.37{X}_{2}{X}_{3}$$

$${Y}_{ \mathrm{A}\mathrm{l}\mathrm{i}\mathrm{z}\mathrm{a}\mathrm{r}\mathrm{i}\mathrm{n}}=+ 1.84{X}_{1}+1.18{X}_{2}+1.40{X}_{3}+2.38{X}_{1}{X}_{2}-1.72{X}_{1}{X}_{3}+0.36{X}_{2}{X}_{3}$$

### Biomass production

**Fig. 1:**
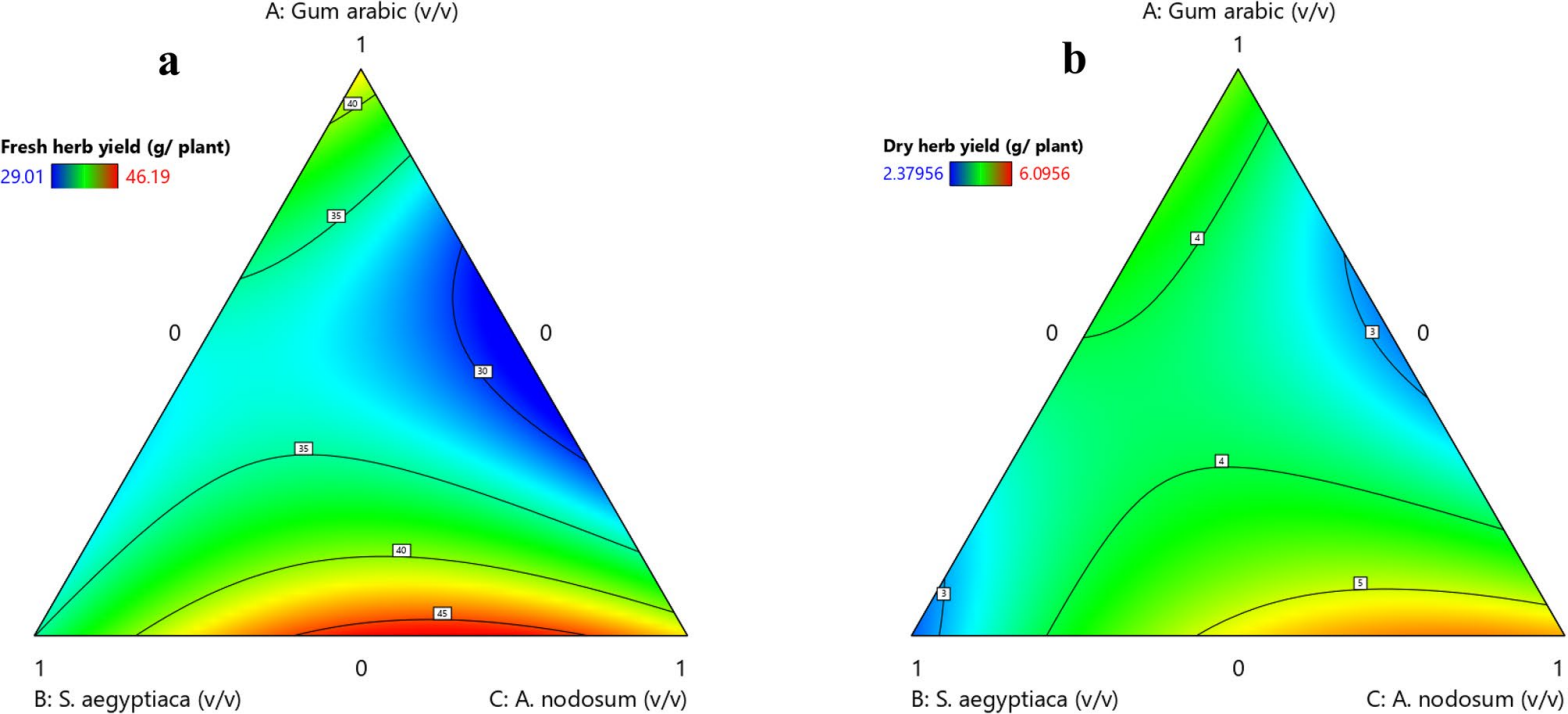
Two-dimensional mixture plots showing the responses of (**a**) fresh herb yield and (**b**) dry herb yield to combinations of the three natural elicitors.

The effects of the three variables on the measured traits were illustrated using two-dimensional response surface plots. In these plots, blue indicates the lowest level of interaction, while red indicates the highest. According to the results, a considerable variation was observed in the effects of the treatments on FHY and DHY, ranging from 29.01 to 46.19 g and from 2.37 to 6.09 g plant^-1^, respectively (Fig. 1a,b). The statistical model was significant for FHY and DHY. The three-way interaction of the treatments for FHY was significant; however, only the AC interaction showed a significant effect on DHY. The highest FHY (46.19 g plant^-1^) was recorded under 0.5 *A. nodosum* and 0.5 *S. aegyptiaca*, whereas the lowest FHY (29.01 g plant^-1^) occurred at 0.5 *A. nodosum* and 0.5 GA. The highest DHY (6 g plant^-1^) was observed in the *A. nodosum* treatment.

### RWC, EL, and SPAD

**Fig. 2:**
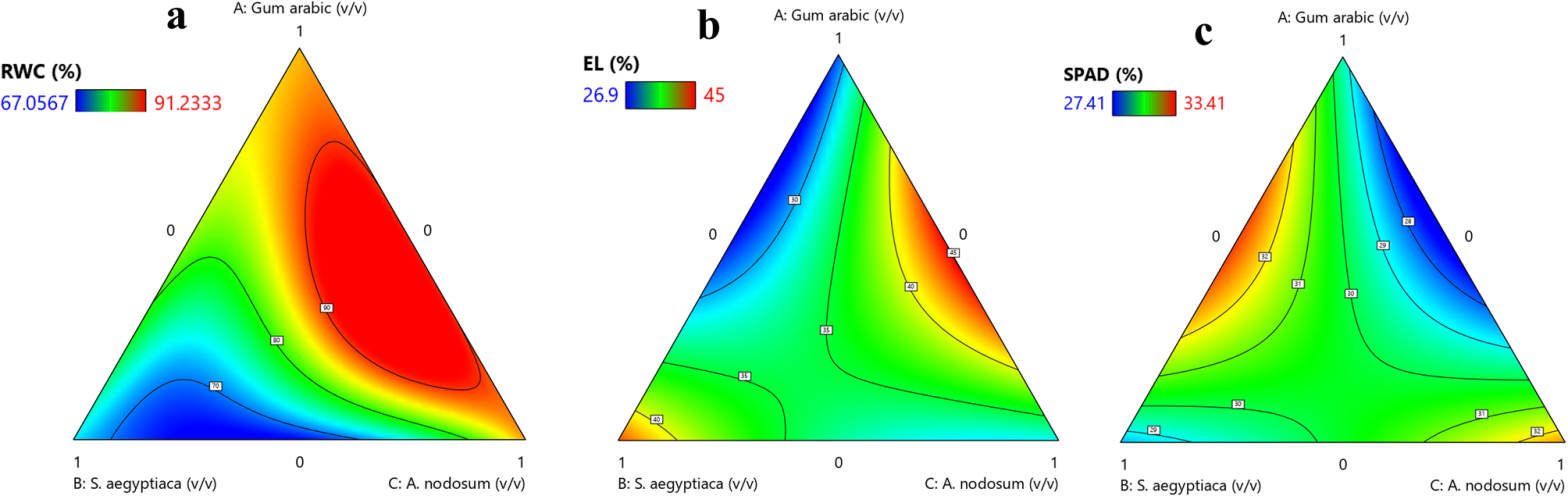
Two-dimensional mixture plots showing the responses of (**a**) relative water content (RWC), (**b**) electrolyte leakage (EL), and (**c**) SPAD values to mixtures of the three natural elicitors.

According to the results, the BC interaction effects on RWC were significant (Fig. 2a). Based on Table 2, the highest RWC (23.91%) was observed under 0.66 *A. nodosum*, 0.16 *S. aegyptiaca*, and 0.16 GA. These findings indicate that the synergistic effects of these treatments increased RWC compared with the individual effect of GA. According to the results, the effects of the model, AB, and AC interactions on EL were significant (Fig. 2b). The highest EL (45%) was recorded in the *S. aegyptiaca* treatment. The combination of 0.5 *S. aegyptiaca* and 0.5 GA exhibited antagonistic effects, resulting in an EL value of 26.9%. However, the combination of 0.5 GA and 0.5 *A. nodosum* affected an EL value of 44%. According to the results, the highest SPAD value (41.33%) was observed with 0.5 GA and 0.5 *S. aegyptiaca* (Fig. 2c). A SPAD value of 33.08% was also recorded for the *A. nodosum* treatment. In contrast, the lowest SPAD value (27.41%) was observed in the *S. aegyptiaca* treatment. These results indicate that the synergistic interaction between *S. aegyptiaca* and GA led to a substantial increase in SPAD values.

### MDA and proline

**Fig. 3:**
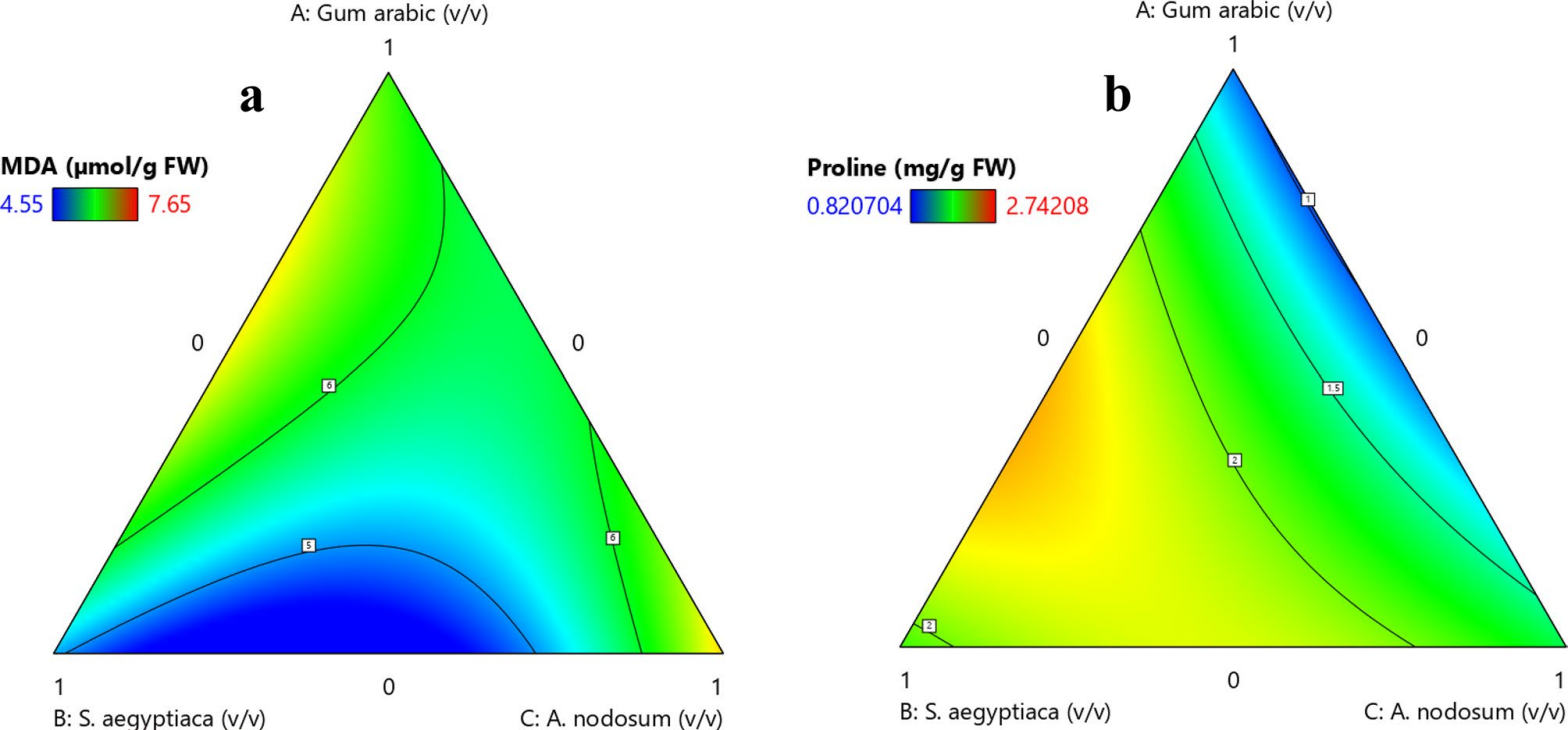
Two-dimensional mixture plots illustrating the responses of (**a**) malondialdehyde (MDA) and (**b**) proline to mixtures of the three natural elicitors.

According to the analysis of variance, the model and the AB and BC interactions were significant for MDA content (Fig. 3a). The highest MDA level (7.65 µmol g⁻^1^ FW) was observed in the *A. nodosum* treatment. The interaction between GA and *S. aegyptiaca* increased MDA to 7.3 (µmol g⁻^1^ FW) compared with the individual effects of GA and *S. aegyptiaca*. However, the interaction between 0.5 *A. nodosum* and 0.5 *S. aegyptiaca* reduced MDA content. The effects of the model and the AB interaction on proline content were also significant. The interaction between 0.5 *S. aegyptiaca* and *A. nodosum* resulted in the highest proline level (2.7 mg g⁻^1^ FW). In contrast, the lowest value (0.82 mg g⁻^1^ FW) was recorded in the GA treatment (Fig. 2b).

### PAL, TPC, TFC, and DPPH

**Fig. 4:**
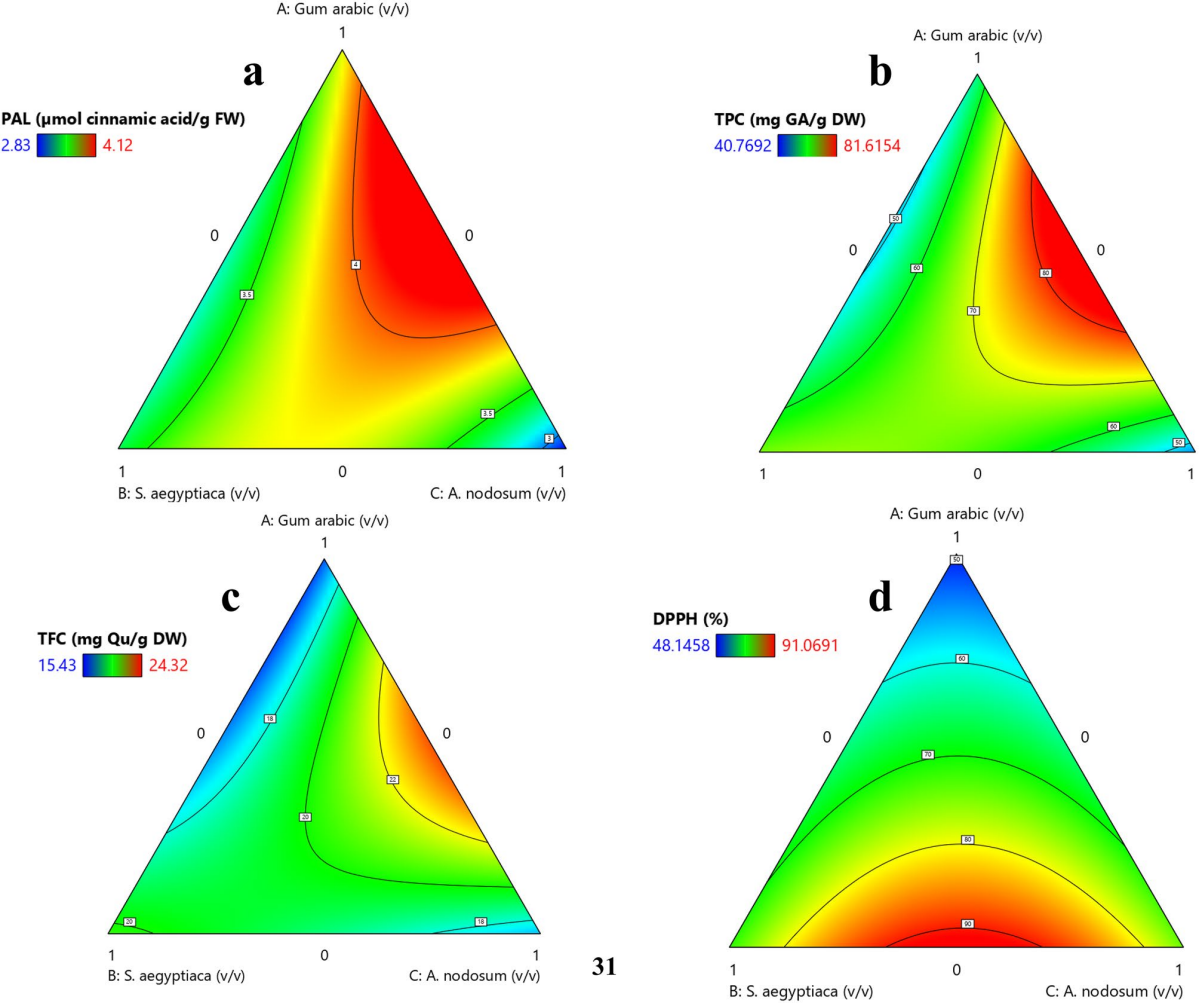
Two-dimensional mixture plots showing the responses of (**a**) phenylalanine ammonia-lyase activity (PAL), (**b**) total phenolic content (TPC), (**c**) total flavonoid content (TFC), and (**d**) DPPH scavenging activity to mixtures of the three natural elicitors.

The analysis table showed that the model and the AC and BC interactions were significant for PAL activity (Table 2). The combination of 0.5 *A. nodosum* and 0.5 GA had a highly significant effect on PAL, resulting in a value of 4.12 (µmol cinnamic acid g⁻^1^ FW) (Fig. 4a). Although equal proportions of the three treatments (0.3) also had a favorable effect on PAL, they had a maximum PAL value of 4.11 (µmol g⁻^1^ FW). The *A. nodosum* alone had the least impact on PAL. However, when combined with other treatments, *A. nodosum* exhibited synergistic interactions, resulting in increased PAL activity.

Based on the analysis of variance table, the model and the BC interaction effects on DPPH scavenging activity were significant (Table 2). The combination of 0.6 *A. nodosum*, 0.16 GA, and 0.16 *S. aegyptiaca* resulted in the highest DPPH scavenging activity (91%) (Fig. 4b). Additionally, the combination of 0.5 *A. nodosum* and 0.5 *S. aegyptiaca* increased DPPH scavenging activity to 88.32%. The lowest DPPH activity (48.14%) was recorded in the GA treatment.

According to the results, the model and the AC interaction effects on TPC and TFC were significant (Table 2). The combination of 0.5 *A. nodosum* and 0.5 GA resulted in the highest increase in TPC, reaching 81.61 (mg GA g⁻^1^ FW) (Fig. 4c). Likewise, an equal proportion of the three treatments (0.3) also caused a substantial increase in TPC, reaching 80.8 (mg GA g⁻^1^ FW). However, the combination of 0.5 *S. aegyptiaca* and 0.5 GA exhibited antagonistic effects on TPC, resulting in a value of 40.76 (mg GA g⁻^1^ FW). The combined effect of 0.5% *A. nodosum* and 0.5% GA produced the highest TFC (24.32 mg Qu g⁻^1^ FW). *S. aegyptiaca* also increased TFC to 21.54 (mg Qu g⁻^1^ FW), compared with the other treatments. The lowest TFC value (15.43 mg Qu g⁻^1^ FW) was observed in the GA treatment (Fig. 4d).

### GPX, CAT, and APX

According to the results, the model significantly affected the activities of GPX, CAT, and APX. The AC interaction significantly affected GPX and APX activities, while the BC interaction significantly affected CAT (Table 2). The highest GPX activity 1.1 (U min⁻^1^ g⁻^1^ FW) was recorded in the leaf of *R. tinctorum*. The equal-ratio combination of the three treatments (0.3) also showed a notable increase in GPX activity (1.02 U min⁻^1^ g⁻^1^ FW). However, the combination of 0.5 *A. nodosum* and 0.5 *S. aegyptiaca* exhibited antagonistic effects on GPX activity 0.54 (U min⁻^1^ g⁻^1^ FW) (Fig. a, b).

**Fig. 5:**
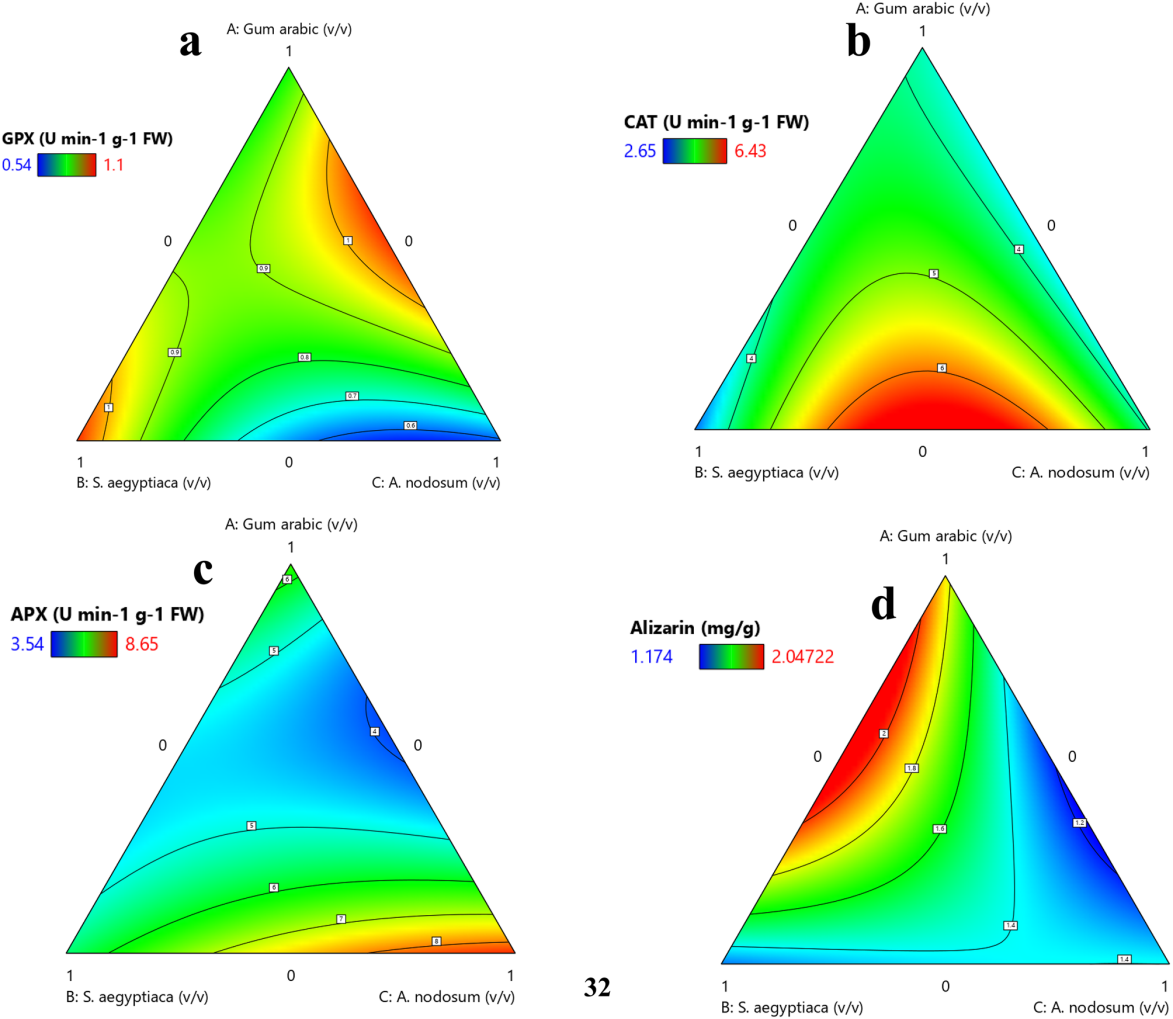
Two-dimensional mixture plots showing the responses of (**a**) guaiacol peroxidase (GPX), (**b**) catalase (CAT), (**c**) ascorbate peroxidase (APX), and (**d**) alizarin to mixtures of the three natural elicitors.

The highest catalase activity was observed under synergistic combinations, including 0.5 *A. nodosum* + 0.5 *S. aegyptiaca* (6.43 U min⁻^1^ g⁻^1^ FW) and 0.16% *A. nodosum* + 0.6% *S. aegyptiaca* (6.01 U min⁻^1^ g⁻^1^ FW), as well as in the GA treatment. The lowest CAT (2.65 U min⁻^1^ g⁻^1^ FW) was recorded in the *S. aegyptiaca* treatment (Fig. 5b). The highest and lowest APX activities were 8.65 (U min⁻^1^ g⁻^1^ FW) in the *A. nodosum* treatment and 3.54 (U min⁻^1^ g⁻^1^ FW) in the combined *A. nodosum* + GA treatment, respectively (Fig. 5d).

### Alizarine

According to the results, the model and the AB and AC interactions were significant for alizarin content (Table 2). The highest alizarin content (2.04 mg g⁻^1^) was obtained from the combination of 0.5% *S. aegyptiaca* and 0.5% GA. In contrast, the combination of 0.5% *A. nodosum* and 0.5% GA resulted in the lowest alizarin content (1.17 mg g⁻^1^) (Fig. 5d).

### Antibacterial activity

**Fig. 6:**
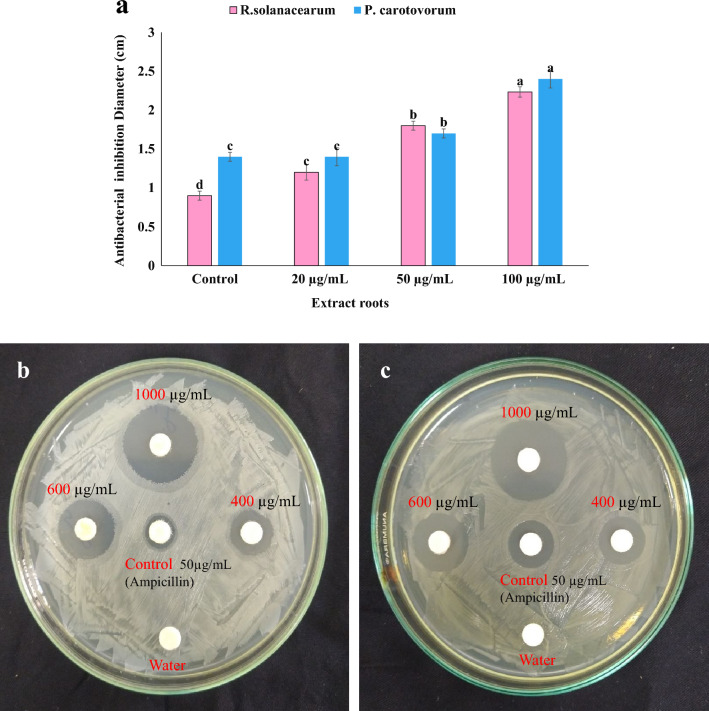
Antimicrobial activity of *R. tinctorum* root extract against (**a**) *Ralstonia solanacearum* and (**b**) *Pectobacterium carotovorum*, and (**c**) Antibacterial property of the extract.

The results indicate a significant effect of *R. tinctorum* root extract against the *P. carotovorum and R. solanacearum* (*P* < 0.01) (Table 3). A concentration of 1000 μL/mL showed the highest inhibitory effect on both bacterial strains (Fig. 6a). For *R. solanacearum*, all tested concentrations of the root extract showed a statistically significant difference compared to the ampicillin control (Fig. 6b). For *P. carotovorum*, the extract at 400 μL/mL did not differ significantly from ampicillin in terms of antibacterial activity (Fig. 6c).

**Table 3:** Analysis of variance of the effect of the extract of roots on *Ralstonia solanacearum* and *Pectobacterium carotovorum.*

*R. solanacearum*			*P. carotovorum*	
S.O.V	df	MS	df	MS
Treatments	3	1.07 **	3	0.66**
Residuals	8	0.015	8	0.025
CV	8.20		9.1	
LSD	0.236		0.29	

*LSD* least significant difference, *SOV* source of variation, *MS* mean square, *Df* degree of freedom, ** Represent significance at *P* ≤ 0.01.

### Molecular docking

**Fig. 7:**
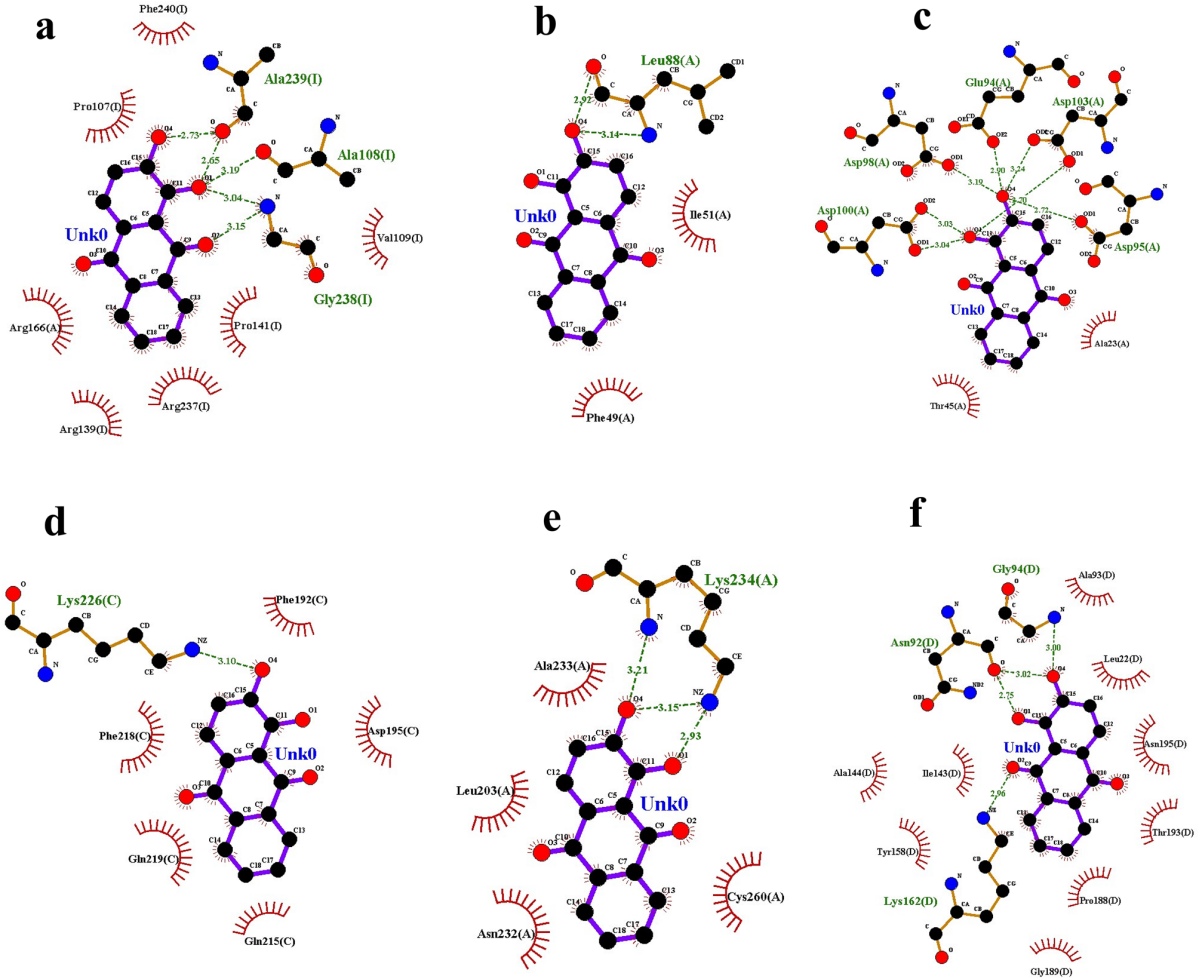
Molecular interaction profiles of alizarin with the active sites of *R. solanacearum* proteins: (**a**) 5NMP, (**b**) 4FDB, (**c**) 1UQX; and *P. carotovorum* proteins: (**d**) 1NX4, (**e**) 4U4B, and (**f**) 4ZA2.

The use of simulation approaches to validate receptor–ligand interactions can provide valuable insights for developing novel therapeutic strategies. Molecular docking is a practical and reliable method for predicting the biological activity of compounds. In this technique, a lower binding free energy indicates a stronger, more favorable affinity between the ligand and the receptor. Moreover, a higher number of molecular interactions generally reflects greater binding stability and potential biological effectiveness. Molecular docking of bioactive compounds against plant pathogens provides a foundation for the design and development of new agents that can inhibit these critical pathogens^41,42^. In the present study, alizarin was docked with the selected target proteins. The docking results are illustrated in Figs. 7 and summarized in Table 4.

**Table 4:** Docking analysis results for multiple ligand combinations on various receptors.

Ligands	Bacterial	Drug target of bacterial species—PDB IDs	Binding Energy (kcal/mol)	Number of Hydrogen Bonds	Interactions	Number of residues to the ligand
Alizarin	*Ralstonia solanacearum*	5NMP	– 8.7	5	Ala239(I), Ala108(I), Gly238(I)	7
		4FDB	– 5.4	2	Leu88(A)	2
		1UQX	– 7.1	7	Asp95(A), Asp103(A), Glu94(A), Asp98(A), Asp100(A)	2
	*Pectobacterium carotovorum*	1N4X	– 6.7	1	Lys226(C)	5
		4UBU	– 6.3	3	Lys234(A)	4
		4ZA2	– 8.7	3	Gly94(D), Asm92(D), Lsy162(D)	9

According to the molecular docking results, the interaction of alizarin with the *R. solanacearum* proteins showed that protein 5NMP exhibited the lowest binding energy (–8.7 kcal mol⁻^1^), whereas 4FDB displayed the highest value (–5.4 kcal mol⁻^1^). Among the proteins, 1UQX formed the most hydrogen bonds (7), followed by 5NMP (5). Additionally, 5NMP established 7 nonpolar interactions with alizarin, indicating that the ligand engages in both polar and hydrophobic contacts with this protein (Fig. 7a- c). For *P. carotovorum*, alizarin showed the strongest interaction with 4ZA2, with a binding energy of –8.7 kcal mol⁻^1^, and formed three hydrogen bonds and nine nonpolar interactions. Alizarin also showed notable binding affinities with proteins 4UBU (–6.3 kcal mol⁻^1^) and 1N4X (–6.7 kcal mol⁻^1^) (Fig. 7d–f).

### Desirability of the assumed model

**Fig. 8:**
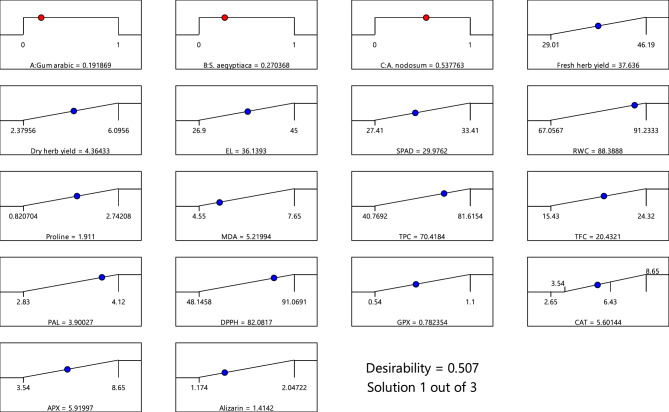
Desirability plot illustrating the optimal proportions of *Ascophyllum nodosum*, *Salix aegyptiaca*, and gum arabic for maximizing physiological, biochemical, and antioxidant responses of *R. tinctorum* under salinity stress.

The results indicate that the optimal mixture consists of 0.19 GA, 0.27 *S. aegyptiaca, and 0.53 A. nodosum* for notable increases in FHY 37.63 (g), DHY 4.36 (g), EL 36.13 (%), SPAD 29.97 (%), RWC (%) 88.38, proline content 1.91 (mg/g FW), MDA 5.21(µmol/g FW), TPC 70.41 (mg GA/g FW), TFC 20.43 (mg Qu/g FW), PAL 3.90 (µmol cinnamic acid/g FW), DPPH scavenging activity 82.08 (%), GPX 0.78 (U min^-1^ g^-1^ FW), CAT 5.60 (U min^-1^ g^-1^ FW), APX 5.91 (U min^-1^ g^-1^ FW), and alizarin 1.41 (mg/g), with desirability of 0.507 (Fig. 8). Consistent with these results, the predictive model showed strong agreement with the experimental data. The mixing ratio of the three natural elicitors can therefore be used to determine the most effective formulation for mitigating the adverse effects of salinity stress in *R. tinctorum*.

### Correlations and PCA analysis of combined data

**Fig. 9:**
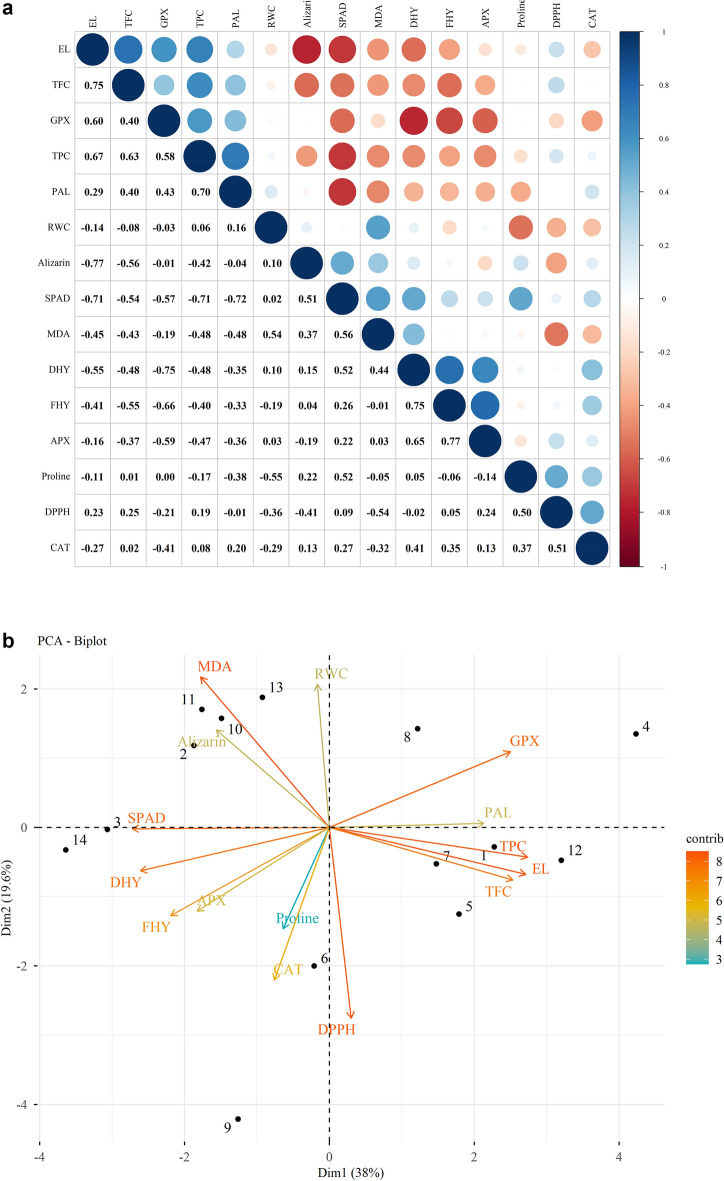
(**a**) Correlation analysis and (**b**) principal component analysis (PCA) based on all evaluated traits. *FHY* Fresh Herb Yield, *DHY* Dry Herb Yield, *SPAD* Spad value chlorophyll content, *RWC* Relative Water Content, *EL* Electrolyte Leakage, *MDA* Malondialdehyde, *TPC* Total Phenolic Content, *TFC *Total Flavonoid Content, *PAL* Phenylalanine Ammonia-Lyase, *GPX* Guaiacol Peroxidase, *CAT* Catalase Peroxidase, APX Ascorbate Peroxidase.

The correlation analysis showed relationships among all measured traits of *R. tinctorum* across all treatments (Fig. 9a). TPC showed strong positive correlations with EL, TFC, and GPX, indicating coordinated metabolic activity. DPPH scavenging activity was positively correlated with phenolic compounds and APX. Alizarin, as a key secondary metabolite indicator, showed negative correlations with TPC, TFC, PAL, and DPPH, but positive correlations with SPAD, MDA, and proline. FHY and DHY were negatively correlated with EL, TFC, GPX, TPC, and DPPH, and positively correlated with SPAD. These correlations indicate that the application of natural elicitors induced coordinated changes across multiple physiological and biochemical traits in *R. tinctorum* under salt stress.

Principal component analysis (PCA) was used to examine the relationships among the measured parameters in response to the applied treatments (Fig. 9b). The first principal component explained 38% of the total variance, while the second explained 19.6%. PC1 was primarily associated with TPC, TFC, PAL activity, and DPPH scavenging activity. PC2 was mainly related to physiological traits, including proline, MDA, alizarin, CAT, and SPAD. Notably, alizarin showed a negative association with FHY and DHY on PC1 and was positioned opposite to TPC, TFC, and antioxidant activity. These results indicate that, under stress conditions, the plant may alter its metabolic pathways and reduce specific metabolites, allocating more energy toward growth.

## Discussion

Salinity stress negatively affects plant growth through ionic toxicity and osmotic imbalance, and it may impair photosynthesis by disrupting carbohydrate allocation. The synergistic application of *A. nodosum*, *S. aegyptiaca*, and GA represents effective strategies for mitigating salinity stress in *R. tinctorum*.

Reduced water potential, decreased tissue water content, and degradation of photosynthetic pigments were identified as major consequences of salinity stress. In line with this study’s results, salt stress decreased biomass in *R. tinctorum*^43,44^. The results showed that the synergistic application of *A. nodosum*, *S. aegyptiaca*, and GA effectively mitigated the adverse effects of salinity on FHY and DHY of *R. tinctorum*. Auxins and cytokinins present in *A. nodosum* enhance cellular metabolism, improve nutrient and organic compound transport, and consequently promote plant growth. Application of *A. nodosum* reduced salt stress in *Solanum lycopersicum*^11^ and *Solanum melongena*^45^ by detoxifying ROS and enhancing nutrient uptake. The principal constituent of *S. aegyptiaca* is salicylic acid, which can positively influence plant growth by regulating physiological processes. SA enhanced plant tolerance to salinity stress by increasing the photosynthetic rate. Salicylic acid may accelerate CO₂ diffusion by increasing leaf cell density, thereby improving growth and photosynthetic performance in *Dianthus superbus* under salt stress^46^. The combined application of the bacterium *Myroid gitamensis*, rice bran, GA, alginate, and tricalcium phosphate significantly increased root length, FW, and DW of wheat seedlings^47^. GA enhances treatment efficiency by forming a protective matrix. GA has high water solubility and a stable glycoprotein structure, which provides improved stability and delivery under stress conditions^47^. The appropriate dosing combination can more effectively enhance plant growth, thereby amplifying the stimulant effects under salt stress.

The content of photosynthetic pigments is a critical determinant of photosynthetic efficiency in plants. In *R. tinctorum*, salinity stress clearly reduced SPAD values. *A. nodosum* functions as a hydration buffer, protecting cellular structures. *A. nodosum* can maintain chlorophyll content by enhancing osmotic potential and increasing nutrient uptake under salinity conditions in *Oryza sativa*^48^. Salicylic acid may mitigate salt‐induced disruptions in the photosynthetic apparatus and enhance chlorophyll biosynthesis^49^. A combination of coffee leaf extract, starch, and GA increases photosynthetic activity in *Solanum lycopersicum* compared to the control^50^. The increased SPAD values observed in treated plants may alleviate the adverse effects of salinity stress in *R. tinctorum*.

The environmental factor of salinity, by inducing oxidative stress, may increase the production of ROS and consequently damage the cellular membrane^51^. MDA and EL are essential indicators of lipid peroxidation under stress conditions. *A. nodosum* and *S. aegyptiaca* may act synergistically to induce plant defense responses and enhance stress tolerance. Salicylic acid can act as a signaling molecule to activate membrane-protective defense responses, thereby reducing salt stress. *A. nodosum* preserved the cell membrane by decreasing reduced MDA and H_2_O_2_ in *Solanum lycopersicum*^52^.

One of the most common biochemical responses of plants to salinity is the accumulation of proline. In the present study, the interaction between *S. aegyptiaca* 0.5 and GA 0.5 showed significantly higher proline accumulation than the other treatments. Increased proline levels help maintain membrane integrity and contribute to osmotic adjustment, thereby mitigating the detrimental effects of salt stress^53^. The increased accumulation of proline and non-structural carbohydrates induced by *A. nodosum* treatment may minimize the damaging effects of salinity stress in plants. *Echium amoenum* seedlings applied to *A. nodosum* significantly increased the proline content under salinity stress^54^. Salicylic acid may enhance plant defense responses by activating proline synthesis. Salicylic acid regulates the expression of stress-responsive genes and the biosynthesis of protective metabolites, enabling plants to better respond to stress^55^.

The natural metabolism of plant cells is disrupted by salinity-induced oxidative stress. Both enzymatic and non-enzymatic compounds increase in plants to help overcome oxidative damage. One of the most effective antioxidant enzymes for mitigating oxidative stress is CAT, which reduces hydrogen peroxide levels in peroxisomes. GPX and APX primarily decrease reactive oxygen species in chloroplasts. The activity of antioxidant enzymes increased under salinity stress in Malus Species^56^ and *Cicer arietinum*^57^. The synergistic application of the treatments resulted in a significant increase in antioxidant enzyme activity. This stimulatory effect may result from the combined influence of micronutrient and macronutrient components across the different treatments. Nevertheless, the specific type and concentration of the applied treatments may elicit antagonistic interactions, potentially mitigating their overall efficacy. In *Lactuca sativa*, treatment with *A. nodosum* increased superoxide dismutase (SOD), CAT, and peroxidase under salt stress compared with the control group^58^. It has been shown that GA increased total peroxidase (POX) and SOD activity, thereby reducing oxidative stress in the anther tissue of *Hordeum vulgare*^59^. The presence of POX within the protein fractions of GA contributes to its antioxidant properties^60^.

TPC and TFC help modulate ROS under salinity stress, supporting plant tolerance. In this study, the synergistic application of *A. nodosum* and GA reduced TPC while enhancing growth, suggesting that the plant allocated more energy to growth rather than secondary metabolite production under stress conditions. The synergistic effects of *A. nodosum* and *S. aegyptiaca* extracts enhanced antioxidant activity. This increased antioxidant capacity can be attributed to the presence of TPC and TFC compounds, which serve as major contributors to the elevated antioxidant potential observed in treated plants. Furthermore, PAL, a key enzyme in the phenylpropanoid pathway, is an essential indicator of plant stress responses^61^. The increased PAL activity observed in *R. tinctorum* may represent a crucial regulatory and adaptive mechanism against salt stress. *A. nodosum* enhancement of DPPH scavenging activity, TPC, and TFC in *Vigna radiata* under salt stress^62^. In Arabidopsis, *A. nodosum* enhanced glutathione S-transferase expression, thereby modulating salt-induced ROS production^63^. The combination of *A. nodosum* and chitosan increased PAL activity and the expression of some defense genes, including those involved in salicylic acid biosynthesis in *Pisum sativum*^64^. GA combined with pomegranate peel increased TFC in *Punica granatum*^65^. The synergistic effects of *S. aegyptiaca* and GA significantly increased alizarin content. In contrast, the interaction between GA and *A. nodosum* reduced alizarin content. It is plausible that the compounds present in *A. nodosum* enhanced growth by changing the pathway of secondary metabolite production. *A. nodosum* altered the biosynthetic pathway of secondary metabolites in the plant^66^.

The natural dye extracted from madder root exhibits antibacterial activity against both Gram-positive and Gram-negative bacteria^67^. Studies have shown that the anthraquinone compound alizarin inhibits the growth of *Cutibacterium acnes*, *Staphylococcus aureus*, and *Candida albicans*^68,69^. Secondary metabolites exhibit antibacterial activity by increasing plasma membrane permeability and inducing ion leakage from bacterial cells^70^. Alizarin exhibits considerable antibacterial activity against *Staphylococcus aureus* and *Listeria monocytogenes*^71^. The presence of OH groups in its structure substantially contributes to its antibacterial properties. Anthraquinone compounds with antibacterial activity may also exhibit lower phytotoxicity compared with synthetic chemicals^72^.

## Conclusion

In this study, a simplex lattice design was used to investigate the synergistic effects of *A. nodosum, S. aegyptiaca*, and GA on the physiological and biochemical responses of *R. tinctorum* under salt stress. This technique can accurately predict synergistic interactions and identify the most effective combinations. The aim was to propose cost-effective and promising methods for assessing the physiological and biochemical traits of *R. tinctorum* under salt stress. Different combinations may produce synergistic effects, and better identification of these components enhances understanding. The combined 0.19 GA, 0.27 *S. aegyptiaca,* and 0.53 *A. nodosum* help *R. tinctorum* alleviate the impacts of salinity stress by improving growth and relative water content, promoting the accumulation of bioactive compounds, stimulating proline accumulation, and increasing antioxidant capacity and alizarin. The application of these natural compounds produces synergistic effects and enhances understanding of plant responses to salt stress. Using root extracts of *R. tinctorum* to evaluate antibacterial activity against *P. carotovorum* and *R. solanacearum* clarified key interactions and supported the research. Applying natural combinations and in silico approaches provides valuable insights for future studies on antibacterial activity. Additionally, utilizing the antimicrobial properties of Madder root extracts could reduce the need for artificial or chemical substances in the development of natural compounds.

## Data Availability

All data relevant to the study are included in the article.
